# Public community knowledge regarding multidisciplinary rehabilitation of spinal cord injury in Lebanon: a cross-sectional study

**DOI:** 10.1186/s12889-025-25287-3

**Published:** 2025-11-18

**Authors:** Nour El Hoda Saleh, Linda Abou-Abbas, Dalia Khachman, Ibrahim Naim, Khaled Mouchref, Salem Hannoun, Samar Rachidi

**Affiliations:** 1 Doctoral School of Sciences and Technology, Lebanese University, Hadat, Lebanon; 2 Clinical and Epidemiological Research Laboratory, Faculty of Pharmacy, Lebanese University, Hadath, Lebanon; 3 Department of Research, Health, Rehabilitation, Integration and Research Center (HRIR), Beirut, Lebanon; 4 Faculty of Medical Sciences, Lebanese University, Beirut, Lebanon; 5 INSPECT-LB (Institut National de Santé Publique Epidémiologie Clinique Et Toxicologie-Liban), Beirut, Lebanon; 6 Laboratoire de Technologie et Instrumentation pour la sante, Faculty of Engineering, Lebanese University, Tripoli, Lebanon; 7 Medical Imaging Sciences Program, Division of Health Professions, Faculty of Health Sciences, American University of Beirut, Beirut, Lebanon

**Keywords:** Spinal cord injury, Rehabilitation, Multidisciplinary care, Lebanon, Community awareness, Knowledge

## Abstract

**Background:**

Research on public community knowledge regarding spinal cord injury (SCI) and its rehabilitation within Lebanon is lacking. The present study aimed to evaluate the level of public knowledge among the Lebanese population concerning SCI and its needed multidisciplinary rehabilitation. In addition, we sought to evaluate the factors associated with the knowledge level.

**Methods:**

An online cross-sectional study was conducted among 1200 Lebanese persons aged 18 years and older. The survey included socio-demographic questions and knowledge levels regarding SCI and rehabilitation.

**Results:**

A total of 1200 participants from the Lebanese community participated in the study, with a mean age of 32.99 ± 11.51 years, and 63.91% were female. Only 63.08% of respondents had heard of SCI, and 39.50% claimed to understand it. Additionally, self-reported knowledge of rehabilitation was low, with 59.17% of participants unfamiliar with the required programs. The mean total knowledge score across all domains was 29.02 ± 14.54 out of 55, indicating an overall knowledge rate of 52.77%. Specific knowledge gaps were identified, particularly regarding the causes of SCI (57.33% had poor knowledge), associated complications (47.42%), differences between physical therapy and rehabilitation (34%), and available rehabilitation services (52.83%). However, participants demonstrated moderate knowledge of SCI rehabilitation goals (60.11%) and lifelong medical and physical care requirements (70.33%). This suggests a generally poor level of knowledge concerning SCI and its rehabilitation among the surveyed Lebanese community members. Factors such as education level (OR = 1.358 (1.047–1.760); *p* = 0.021), working in the healthcare field (OR = 4.787 (3.191–7.182); *p* < 0.0001), and previous consultation with rehabilitation specialists significantly influenced knowledge scores (OR = 2.518 (1.899–3.338); *p* < 0.0001).

**Conclusion:**

This study highlights the lack of awareness regarding SCI and its essential rehabilitation among the Lebanese population. Significant gaps were identified in knowledge levels, particularly regarding the understanding of SCI complications and the need for multidisciplinary rehabilitation. Educational interventions and awareness campaigns are warranted to improve community support and enhance outcomes for individuals living with SCI in Lebanon.

**Supplementary Information:**

The online version contains supplementary material available at 10.1186/s12889-025-25287-3.

## Background

Spinal cord injury (SCI) is a serious neurological disorder with an increasing global incidence, with 0.9 million new cases in 2019 [1]. The impact of SCI extends beyond physical impairments to include significant emotional, social, and economic challenges [2]. It also involves various secondary health conditions leading to morbidity, early aging, and community participation restrictions [3]. Multidisciplinary rehabilitation, which includes medical, physical, psychological, and social interventions, is essential for enhancing the quality of life and functional outcomes for individuals with SCI [4–6]. This approach aims to restore community reintegration in individuals by addressing secondary complications in various body systems, such as urinary tract infections, pressure ulcers, orthostatic hypotension, pulmonary and cardiovascular problems, and depressive disorders [7, 8].

Community awareness is essential in public health and prevention [9]. National and local awareness projects are recommended to identify and address barriers at the level of individuals, communities, and systems of care levels [10]. This approach is particularly important for individuals with SCI, as a lack of knowledge has been reported as a barrier to adaptation [11]. Insufficient understanding of SCI and its rehabilitation needs can lead to stigmatization, social isolation, and inadequate support systems, further hindering recovery and community reintegration [12, 13]. Policymakers should prioritize raising public awareness of SCI care to address these barriers, improve outcomes, and improve health rehabilitation services [14]. However, research on community awareness and attitudes toward SCI rehabilitation is lacking globally. Recent studies highlight the importance of public awareness in addressing spinal cord injuries. For instance, a study in Makkah, Saudi Arabia, found that 53.8% of participants had an acceptable awareness level of cervical spine injuries [15]. For instance, two studies in Iran highlighted different factors, including traditional and suppressive attitudes, affecting healthcare and rehabilitation plans for SCI individuals [13, 16].

In Lebanon, specific data on SCI prevalence and incidence are limited, reflecting a broader scarcity of research on community awareness and attitudes towards SCI rehabilitation in the Arab world. However, the World Health Organization’s Rehabilitation Need Estimator indicates that approximately 1.6 million individuals in Lebanon could benefit from rehabilitation services, highlighting a substantial unmet need [17]. Despite this, access to rehabilitation remains severely limited, with services predominantly provided by the private sector and concentrated in urban areas, leaving rural regions underserved [18]. Financial barriers are significant, exacerbated by the ongoing economic crisis, with out-of-pocket spending accounting for nearly 50% of total healthcare costs. Moreover, there is a narrow understanding of rehabilitation, often perceived as limited to physiotherapy, while multidisciplinary approaches—including occupational therapy, speech therapy, and psychosocial support—are frequently neglected [18]. These systemic challenges highlight the need for inter-ministerial coordination, integration of rehabilitation into the national health system, and targeted awareness campaigns to improve accessibility, affordability, and utilization of rehabilitation services for individuals with SCI and other disabilities [17].

A critical component in addressing these barriers is improving community awareness and understanding of SCI rehabilitation. This highlights the necessity of evaluating and improving public knowledge and attitudes in Lebanon to ensure that rehabilitation services are utilized effectively and tailored to the needs of individuals with SCI. Thus, this study was conducted with the aim of evaluating the level of public knowledge among the Lebanese population concerning SCI and its multidisciplinary rehabilitation. By assessing the current state of knowledge and identifying the most significant gaps, this research seeks to provide a foundation for developing targeted strategies to raise awareness and improve the utilization of rehabilitation services. Ultimately, the goal is to enhance the quality of life and rehabilitation outcomes for individuals with SCI in Lebanon, contributing to a more informed and supportive community.

## Methods

### Study design and population

A cross-sectional study was conducted between December 2023 and April 2024 among Lebanese community members across all governorates of Lebanon. Eligible participants included Lebanese individuals aged 18 years and above residing in Lebanon’s eight governorates. Individuals suffering from any intellectual disorder or cognitive delay, those who could not read or understand the Arabic language, and persons with a personal history of SCI were excluded from the study.

### Ethical considerations

Consent was obtained from subjects before starting the data collection, and they were informed that participation was voluntary. No personal or confidential information was collected. The study adhered to the research ethics guidelines of the Declaration of Helsinki and received approval from the Institutional Review Board at the Health, Rehabilitation, Integration, and Research Center in Beirut, Lebanon, on December 29, 2023 (reference number HRIR.P020.05.12/2023).

### Sample size

The sample size was calculated using the online Raosoft sample size calculator. The sample size was calculated based on an estimated response rate of 50%, a 95% confidence interval, a 5% margin of error, and a Lebanese population size of 4,842,000 inhabitants based on the latest Lebanese census data [19]. Taking into account the stratified sampling method, the minimum required sample size of 1118 participants was calculated.

### Sampling strategy

For sampling, a stratified convenient sampling method was employed, selecting participants from each of Lebanon’s eight governorates based on population size data from 2019 [19]. The number of participants in each governorate was calculated using a probability-proportion-to-size sampling method based on the data provided by the Central Administration of Statistics in 2019 of each governorate population size [19]. Details regarding the number of participants collected from each governorate are provided in the additional file.

### Questionnaire

An online survey was designed to evaluate the respondents’ level of knowledge of SCI and its rehabilitation. The questionnaire was prepared in Arabic after an extensive review of the literature on SCI complications and the principles of rehabilitation [8, 20–22]. Items were initially generated based on key themes identified in the literature, ensuring coverage of essential aspects of SCI knowledge and rehabilitation.

To establish content validity, a panel of experts, including physiotherapists, a physical medicine and rehabilitation doctor, and rehabilitation researchers, reviewed the questionnaire. They assessed the relevance, clarity, and comprehensiveness of each item. Based on their feedback, modifications were made to refine question wording, improve clarity, and eliminate redundancy.

The first section of the questionnaire collected socio-demographic data, including age, gender, marital status, educational level, occupation, and family income. Furthermore, information regarding their family history of SCI and whether they knew any family members or friends who had a previous SCI was gathered in this section. The second section assessed the self-reported level of knowledge of SCI, and its resulting complications. The third section included questions on knowledge regarding rehabilitation in general, its goals in SCI, and the source of information. In addition, specific questions on the different rehabilitation professions required for SCI rehabilitation were asked. Questions on the perceived need for knowledge about rehabilitation were also included at the end of the questionnaire. Some items involved multiple-choice selections, yet the majority required respondents to choose between “Yes”, “No”, or “Don’t know” for each item. Items with correct answers were scored “1” and incorrect or “Don’t know” answers were scored 0. Each section’s score was the sum of items within that section. There were 10 questions on the causes of SCI, 20 on complications, 2 single items on knowledge of long-term care and the difference between rehabilitation and physical therapy, 9 questions on SCI Rehabilitation Goals, and 14 on Knowledge of SCI rehabilitation services. The overall knowledge score was calculated by summing the six section scores. The maximum possible score was 55. Participant scores were then converted to a percentage by averaging their total score over 100. To categorize participants’ knowledge levels Bloom’s cut-off criteria were applied [23]. This standardized method classifies knowledge scores into two categories: “good knowledge” (≥ 60%) and “poor knowledge” (< 60%). Bloom’s cut-off is widely used in health education and Knowledge, Attitude, and Practice (KAP) studies as a practical and validated approach to interpret knowledge survey scores and identify gaps. For instance, similar cutoff points have been applied in health education research to assess knowledge about chronic diseases, preventive practices, and rehabilitation [24–27].

### Pilot testing

To ensure clarity, comprehensibility, and comprehensiveness, the questionnaire was revised by a physical medicine and rehabilitation doctor, a rehabilitation nurse, two physical therapists experienced in SCI rehabilitation, and a clinical psychologist. Subsequently, the questionnaire was piloted with 20 individuals with SCI, and adjustments were made based on participant feedback regarding any ambiguous questions or statements. On average, participants took approximately 8 min to complete the survey.

### Data collection

Eligible participants were asked to participate in the survey through social media and online advertisements via an online survey via a link to “Google Forms” software. An invitation letter concerning the survey was sent with the link to the questionnaire via social media, including information describing the survey and asking for the voluntary participation of the Lebanese community members. Participants were initially presented with an introductory survey interface upon accessing the survey link. This interface provided a comprehensive overview of the study objectives, along with clearly stated inclusion and exclusion criteria. Individuals were instructed to self-assess their eligibility based on these criteria and to discontinue the survey if they met any exclusion conditions. Before proceeding to the questionnaire, participants were required to confirm their voluntary participation, with an emphasis on confidentiality in data collection, storage, and informed consent.

### Statistical analysis

The collected data was analyzed with the Statistical Package for Social Sciences software (SPSS) version 26. Responding to all the questions was mandatory; there was no missing data. The socio-demographic characteristics and knowledge scores of the participants were described using the mean and standard deviation for continuous variables and frequencies with percentages for categorical variables. Chi-square was used to determine the association between different variables including age, gender, occupation, marital status, living with someone with SCI, having a friend with SCI, previous consultation with a rehabilitation specialist, self-rating of knowledge of rehabilitation, and poor (< 60%) and good (>60%) knowledge [23]. The variables in bivariate analysis with *p*-values < 0.20 were entered into the model of binary logistic regression analysis to delineate factors associated with poor or good knowledge. Adjusted odds ratios and 95% confidence intervals were reported. The final logistic regression model was reached after confirming the adequacy of our data using the Hosmer and Lemeshow test [28]. A *p*-value less than 0.05 was considered significant.

## Results

### Baseline characteristics of the study participants

Table 1 shows the baseline characteristics of the study participants. Between December 2023 and April 2024, a total of 1200 Lebanese community members participated in this study. Their ages ranged between 18 and 70 years, with a mean of 32.99 ± 11.51 years. Overall, 63.91% were female, and the majority of them were well-educated, with 38.84% holding a bachelor’s degree and 28.26% having postgraduate qualifications. In terms of employment, 59.75% were employed, and 46.33% of the participants were married. Regarding monthly family income, 31.33% earned more than $800, while 23.83% earned less than $300 per month. Of the 1200 participants, only 4.33% (*n* = 52) reported living with a person with SCI, and 12.42% (*n* = 149) reported having a friend or relative with SCI.

**Table 1:** Baseline characteristics of participants (*n* = 1200)

Variable	Frequencies	Percentage (%)
Gender		
Male	433	36.09
Female	767	63.91
Educational Level		
Elementary (< 6years)	9	0.76
Middle or High School (6–12 years)	386	32.17
College or University (12–16 years)	466	38.84
Postgraduate (> 16 years)	339	28.26
Employment status		
Employed	717	59.75
Unemployed	483	40.25
Occupation		
Employee/Office Worker	184	15.33
Healthcare professional	198	16.50
Manual Workers (Construction worker/maintenance worker/cleaner)	18	1.50
Education	129	10.75
Other	188	15.67
Unemployed	483	40.25
Marital Status		
Married	556	46.33
Unmarried	605	50.41
Divorced	24	2.01
Widowed	15	1.25
Family Income		
100–300$	286	23.83
300–500$	293	24.42
500–800$	245	20.42
> 800$	376	31.33
Living with a person having SCI		
Yes	52	4.33
No	1148	95.67
Having a friend or relative with SCI		
Yes	149	12.42
No	1051	87.58

	Mean ± SD	
Age	32.99 ± 11.51	

*SD* standard deviation

### Self-rated knowledge of SCI and its rehabilitation

The participants’ responses on the recognition of SCI and its rehabilitation are reported in Table 2. Most participants (63.08%) indicated that they had heard of SCI when asked if they had any prior knowledge of the condition. On the other hand, in terms of understanding SCI, only 39.50% of the 1200 participants claimed they knew what SCI is. Self-rated perceived knowledge of rehabilitation was also limited, with only 17.67% reporting very good knowledge. This self-reported lack of knowledge extended to familiarity with health and rehabilitation services for individuals with SCI, as 77.25% of respondents expressed not being familiar with such programs. As sources of information on SCI rehabilitation, respondents primarily relied on social media (29.16%), personal experiences involving friends or relatives (16.75%), and information from education (12.50%), while 20.5% admitted to lacking knowledge about SCI rehabilitati*o*n.

**Table 2:** Self-rated knowledge about SCI and rehabilitation

Information on Knowledge Item	Frequency	Percentage
Have you ever heard of spinal cord injury?		
Yes	757	63.08
No	246	20.5
Maybe	197	16.42
Do you know what SCI is?		
Yes	474	39.50
No	418	34.83
Maybe	308	25.67
How do you rate your level of knowledge in the field of rehabilitation?		
Weak	695	57.92
Average	239	24.41
Very Good	212	17.67
Do you consider yourself familiar with health and rehabilitation services or programs for people with spinal cord injuries?		
Yes	273	22.75
No	710	59.17
Maybe	217	18.08
Self-reported Perceived knowledge of comprehensive multidisciplinary treatment in spinal cord injury rehabilitation		
Yes	230	19.20
No	970	80.80
Have you ever consulted a rehabilitation specialist or visited a rehabilitation center previously?		
Yes	318	26.50
No	841	70.08
Maybe	41	3.42
Perceptions towards support for people with spinal cord injury in Lebanese Society		
Access to rehabilitation care		
No	712	59.33
Yes	253	21.08
I Don’t Know	235	19.59
Accessibility in terms of parking, ramps, working elevators, restroom access, and wide doorways and hallways.		
No	852	71.00
Yes	206	17.17
I Don’t Know	142	11.83
Education		
No	667	55.59
Yes	334	27.83
I Don’t Know	199	16.58
Employment		
No	845	70.42
Yes	153	12.75
I Don’t Know	202	16.83
Community integration		
No	711	59.25
Yes	257	21.42
I Don’t Know	232	19.33
Perceived need for knowledge and awareness about rehabilitation treatments within Lebanese society.		
Strongly disagree	3	0.25
Disagree	4	0.33
Neutral	49	4.08
Agree	245	20.42
Strongly agree	899	74.92
Intent for Information-seeking		
Very unlikely	12	1.00
Unlikely	25	2.08
Neutral	98	8.17
Likely	529	44.08
Very likely	536	44.67

Self-reported perceptions of support for people with SCI within Lebanese society varied across different domains. Concerning access to rehabilitation care, 59.33% of respondents indicated a lack of access for individuals with SCI, while 71% reported inadequate accessibility in terms of parking, ramps, elevators, and restroom access. Additionally, 55.59% and 70.42% of respondents expressed a lack of access to educational opportunities and employment support, respectively, for people with SCI. Similarly, for community integration, 59.25% perceived insufficient support. A total of 74.92% agreed on the need for awareness about rehabilitation within Lebanese society. Furthermore, 52.25% reported a likely intent to seek information about spinal cord injuries and rehabilitation after this survey.

### Evaluation of awareness regarding SCI and its rehabilitation

The evaluation of community knowledge regarding SCI and its rehabilitation is presented in Table 3. The results revealed varying levels of knowledge across different aspects of SCI among participants. The majority of respondents exhibited poor knowledge in several areas. Specifically, poor knowledge was observed in response to questions concerning the causes of SCI (57.33%), complications associated with SCI (47.42%), differences between physical therapy and rehabilitation (34%), and knowledge of SCI rehabilitation services (52.83%). However, participants demonstrated moderate knowledge of SCI rehabilitation goals, resulting in a mean score of 5.41 ± 3.03 out of 9, with an overall knowledge rate of 60.11% and knowledge of lifelong required medical and physical care for individuals with SCI (70.33%). Overall, across all domains, participants achieved a mean total score of 29.02 ± 14.54 out of 55, corresponding to a knowledge rate of 52.73%, indicating a poor level of knowledge. However, these scores primarily reflect theoretical knowledge rather than practices in accessing or utilizing rehabilitation services.

**Table 3:** Frequencies and percentages of the participant’s awareness regarding SCI and its rehabilitation

Knowledge	Correct Responses		Wrong Responses		I Don’t Know		Average Score
	*n*	%	*n*	%	*n*	%	Mean ± SD
Causes of SCI	688	57.33	124	10.33	388	32.34	5.73 ± 3.24 (0–10)
Complications of SCI	569	47.42	167	13.91	464	38.67	9.29 ± 5.99 (0–20)
Knowledge of the life-long required medical and physical care for individuals with SCI	844	70.33	44	3.67	312	26.00	0.70 ± 0.46 (0–1)
knowledge of the difference between physical therapy and rehabilitation	408	34.00	296	24.67	496	41.33	0.34 ± 0.47 (0–1)
Knowledge of SCI Rehabilitation Goals	729	60.75	159	13.25	312	26.00	5.41 ± 3.03 (0–9)
Knowledge of SCI rehabilitation services	634	52.83	263	21.92	303	25.25	7.53 ± 4.47 (0–14)
Total Score							29.02 ± 14.54 (0–55) 52.73%

### Source of information regarding SCI rehabilitation

**Fig. 1:**
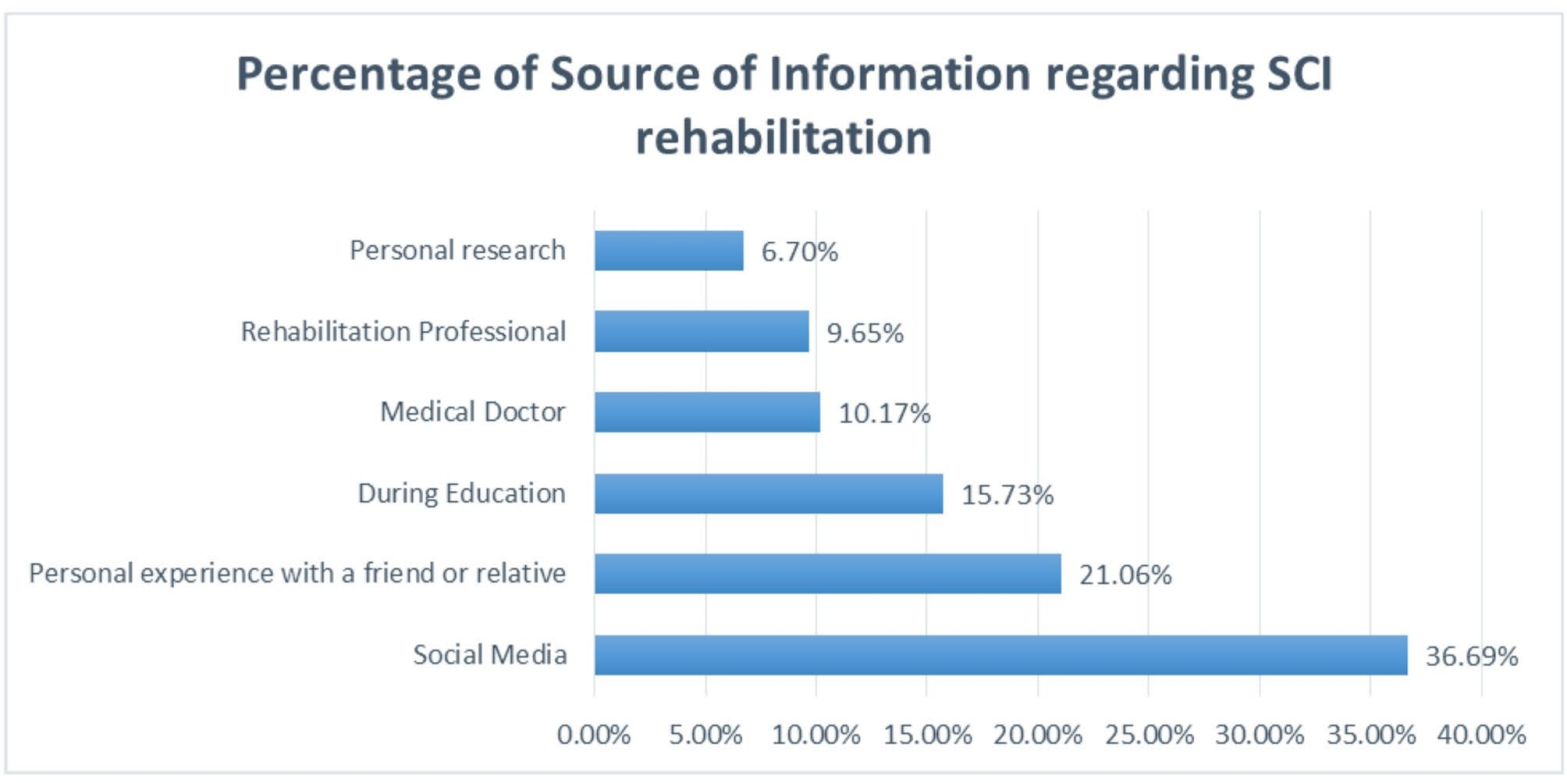
Sources of information regarding SCI rehabilitation

The primary sources of information on SCI rehabilitation among the participants were social media platforms, which accounted for 26. 69% of the responses. This was followed by personal experience with a family member or friend having SCI, which was reported by 21.06% of the participants. In contrast, only 9.65% of the participants obtained information from a rehabilitation professional. A detailed description of the sources of information regarding SCI rehabilitation is presented in Fig. 1.

### Factors associated with good knowledge of SCI and its rehabilitation

Table 4 illustrates variables significantly associated with knowledge levels in both bivariate and multivariate analyses. In bivariate analysis, variables such as educational level (*p* < 0.0001), occupation (*p* < 0.0001), living with someone with SCI (*p* = 0.039), having a friend or relative with SCI (*p* = 0.034), previous consultation with a rehabilitation professional (*p* < 0.0001), and self-assessed knowledge level (*p* < 0.0001) were found to be associated with a good knowledge score. In the binary logistic regression model, being female (OR = 1.341, 95% CI: 1.037–1.734; *p* = 0.025), having a university degree (OR = 1.358, 95% CI: 1.047–1.760; *p* = 0.021), working in a health profession (OR = 4.787, 95% CI: 3.191–7.182; *p* < 0.0001), previous consultation with a rehabilitation professional (OR = 2.518, 95% CI: 1.899–3.338; *p* < 0.0001), and self-rating knowledge as good (OR = 5.03, 95% CI: 3.20–7.89; *p* < 0.0001) were significantly associated with a good knowledge score compared to their respective counterparts.

**Table 4:** Factors associated with good knowledge of SCI and its rehabilitation

Factors	Poor Knowledge *n* (%)	Good Knowledge *n* (%)	Bivariate Analysis *p*-value	Multivariate Analysis	
				Adjusted OR (95% CI)	*p*-value
Age range					
<30 years	314	283	0.90		
>30 years	315	288			
Gender					
Male	238	195	0.18	1.00	0.02
Female	391	376		1.34 (1.03–1.73.03.73)	
Marital Status					
Married	288	268	0.69		
Unmarried	341	303			
Educational Level					
<12 years of education	239	156	<0.0001	1.00	0.02
>12 years of education	390	415		1.35 (1.04–1.76.04.76)	
Occupation					
Not Healthcare	594	411	<0.0001	1.00	<0.0001
Health professional	35	160		4.787 (3.19–7.18.19.18)	
Living with SCI individual					
No	609	539	0.03	1.00	0.15
Yes	20	32		1.68 (0.82–3.45.82.45)	
SCI relative or friend					
No	563	488	0.03	1.00	0.69
Yes	66	83		0.91 (0.58–1.43.58.43)	
Previous consultation with a rehabilitation specialist					
No	515	326	<0.0001	1.00	<0.0001
Yes	114	245		2.51 (1.89–3.33.89.33)	
Self-rating of the level of knowledge of rehabilitation					
Weak	459	236	<0.0001	1.00	<0.0001
Average	131	162		2.48 (1.57–3.92.57.92)	
Good	39	173		5.03 (3.20–7.89.20.89)	

## Discussion

This study investigated the level of public knowledge among the Lebanese population concerning SCI and its needed multidisciplinary rehabilitation. The results revealed poor community knowledge of rehabilitation following SCI in Lebanon. It reported the responses of 1200 Lebanese community members across its eight governorates, showing that the accuracy of most knowledge items was less than 60%, averaging 52.77%. These findings underscore the insufficient knowledge of SCI and its requisite rehabilitation within the Lebanese community [29].

Notably, only 47.42% of respondents accurately identified complications associated with SCI, which is indicative of a significant knowledge gap in understanding secondary issues. These results align with prior research examining SCI awareness among the general public, individuals with SCI, and caregivers, emphasizing the need for educational interventions to address these gaps [30–34].

In our study, 70.33% of respondents indicated that SCI necessitates lifelong medical and physical care, indicating a greater knowledge level than that reported in a previous study conducted in Saudi Arabia, where only 55.1% of respondents recognized the potential need for lifelong medical and physical management for individuals with cervical spine injuries [22]. In addition, only 34% of participants were able to differentiate between physical therapy and rehabilitation, while 52.83% demonstrated knowledge of SCI rehabilitation services. This lack of knowledge can be attributed to the underdevelopment of SCI rehabilitation services in low- to middle-income countries, where physical therapy and exercise are often regarded as the sole forms of rehabilitation [35]. These findings align with the scarcity of multidisciplinary rehabilitation centers in Lebanon, where both private and public organizations tend to prioritize physiotherapy as the only essential rehabilitation modality [36].

Poor knowledge of SCI rehabilitation was evident in understanding the necessary rehabilitation services and in recognizing the need for a multidisciplinary team. While no studies have explored knowledge levels regarding SCI rehabilitation, only one study focused on awareness of vocational rehabilitation in individuals with SCI; 77% of participants were unaware of vocational rehabilitation, and only 44% had accessed such services [37]. Therefore, while the classification of knowledge levels provides a quantitative measure of awareness, it does not necessarily translate into real-world competence. A good knowledge score indicates familiarity with SCI rehabilitation but does not ensure the ability to navigate healthcare systems, advocate for care, or make informed decisions. Structural barriers such as financial constraints, limited rehabilitation facilities, and societal stigma further hinder access, in addition to a lack of awareness of available services [38, 39], particularly in underserved communities. Public misconceptions can reinforce discrimination, limit social and economic opportunities for individuals with SCI, and contribute to inadequate policy support and funding for rehabilitation programs [40]. In other terms, ​health literacy is a crucial determinant of health outcomes for individuals with SCI [41]. Inadequate health literacy has been linked to poorer physical and mental health, functional limitations, increased risks of comorbidities, and higher healthcare expenses [42]. Factors such as access to care, social support, and self-efficacy may mediate the relationship between health literacy and quality of life in individuals with chronic conditions [43]. Future research should explore these mediating factors to better understand how health literacy impacts decision-making, service utilization, and long-term rehabilitation outcomes, thereby informing targeted interventions to enhance quality of life of individuals with SCI.

Social media emerged as the primary source of information about SCI rehabilitation (36.69%), and personal experiences were reported by 21.06%. This is a reasonable result given the widespread use of the Internet and smartphones. This finding aligns with a prior study on knowledge about COVID-19 in Arabic countries, where social media dominated information sources [44]. On the positive side, social media platforms provide accessible, real-time information and foster communities where individuals with SCI can share experiences, seek advice, and find emotional support [45]. This can empower patients by increasing their knowledge about SCI management, rehabilitation options, and emerging treatments. Additionally, social media enables the rapid dissemination of health-related updates, connecting users with healthcare professionals, advocacy groups, and peer networks that may otherwise be difficult to access [45]. However, the drawbacks of social media as a health information source cannot be overlooked. Unlike established medical resources, social media often lacks credibility and scientific review, leading to the spread of misinformation or unverified claims, which can misguide patients and potentially harm their health outcomes [46]. Furthermore, the overwhelming volume of information on social media can make it challenging for users to discern credible sources from unreliable ones. In the context of SCI, where accurate and tailored information is critical, reliance on unverified social media content may lead to unrealistic expectations or inappropriate self-management strategies. Therefore, while social media can be a valuable tool for education and support, it should be used cautiously and complemented with information from trusted healthcare providers and evidence-based resources [47].

The study also identified factors influencing knowledge levels. Individuals with higher education levels exhibited significantly higher knowledge scores, which can be justified by the relationship between health literacy and educational attainment [48, 49]. Furthermore, personal connections to individuals with SCI significantly impacted knowledge. Indeed, personal connections to individuals with SCI may facilitate direct engagement with rehabilitation processes and services, as people may actively participate as caregivers, accompany their loved ones to medical appointments, and seek out information about available rehabilitation programs and resources. Through these experiences, individuals gain knowledge of SCI, the required rehabilitation services, and multidisciplinary care, thereby contributing to their enhanced knowledge levels. In addition, the fact that the knowledge scores of participants align well with their self-reported rating of knowledge provides evidence for the construct validity of the instrument (OR = 5.03, 95% CI: 3.20–7.89; *p* < 0.0001). This alignment suggests that participants were likely to respond honestly when unsure about a topic rather than guessing or fabricating answers based on limited knowledge. Such transparency strengthens the questionnaire’s validity by reducing potential biases that could arise from inaccurate or speculative responses. To the best of our knowledge, this is the first study conducted in Lebanon and the Arab world investigating the awareness of rehabilitation in SCI. Given the scarcity of research on rehabilitation-related topics in the Arab region, this study fills a significant gap in the literature and provides valuable insights into rehabilitation knowledge levels within the Lebanese general community. However, the findings of the present study should be interpreted within the context of its limitations, notably the reliance on self-reported information, which could introduce response bias. Moreover, the cross-sectional design of the study limits our ability to establish causal relationships between variables and only provides a snapshot of SCI rehabilitation knowledge at a specific point in time. One limitation of this study relates to the recruitment method, where participants were recruited via an online survey relying on self-selection and self-screening to apply inclusion and exclusion criteria. Although an introductory section clearly outlined eligibility requirements and instructed participants to self-assess prior to completing the questionnaire, this process cannot guarantee full compliance or verification of exclusion conditions such as cognitive impairments or prior SCI history. This is a well-recognized limitation of anonymous, self-administered web-based surveys, where direct clinical screening or interviewer verification is not feasible. Therefore, some individuals who did not strictly meet al.l criteria may have participated, potentially introducing bias. Additionally, the unemployment rate in our sample (40%) was considerably higher than the national average of approximately 29.6% reported by the Central Administration of Statistics and the International Labour Organization in January 2022. This disparity likely reflects volunteer bias, recruitment channels, and socio-demographic factors that could limit the representativeness of our sample. While the sample size was calculated to be representative of the population, this calculation alone does not ensure that all sample characteristics, including employment status, perfectly reflect the broader population. Specifically, relying on online recruitment through social media may have introduced selection bias by over-representing individuals with better internet access, higher digital literacy, or particular demographic profiles, while under-representing older adults, lower socioeconomic groups, and rural populations [47]. Furthermore, online surveys frequently attract respondents with higher education levels and women, which may further skew sample representativeness [50].

In light of these factors, findings related to employment and other socio-demographic variables should be interpreted cautiously. Future research would benefit from incorporating more robust recruitment strategies, including targeted sampling, community outreach, and in-person data collection, as well as applying statistical weighting to adjust for sample imbalances and improve generalizability.

Additionally, the study’s focus on Lebanon restricts the generalizability of findings to other cultural or socio-economic contexts. Moreover, it is important to consider that external factors, such as media campaigns, public health initiatives, and other ongoing awareness programs during the study period, may have influenced participants’ knowledge levels. Although the questionnaire included questions about sources of information on SCI rehabilitation, it did not specifically assess exposure to such initiatives. Future research could explore this aspect by incorporating items that evaluate participants’ engagement with educational campaigns or other informational resources to better understand their impact on knowledge acquisition.

In addition, while the binary categorization of knowledge levels using Bloom’s cutoff criteria offers practical utility for benchmarking and comparison, it is important to acknowledge that this approach may not fully capture the complexity and nuances of SCI knowledge. By categorizing knowledge as either “good” or “poor,” subtle variations in understanding and context-specific insights may be overlooked. Nevertheless, the use of this method aligns with established educational and health research practices, providing a standardized framework that facilitates the meaningful interpretation and application of findings [24, 26, 29]. Future studies may consider incorporating more nuanced assessment tools or mixed-method approaches to better reflect the multifaceted nature of SCI knowledge while retaining the practical advantages of cutoff-based categorization. Lastly, while efforts were made to ensure the survey’s validity and reliability, the absence of external references or benchmarks specific to SCI rehabilitation knowledge in Lebanon further underscores the need for caution in interpreting and applying the study’s findings.

Despite these limitations, our study revealed a significant lack of awareness among the Lebanese population regarding SCI and its requisite multidisciplinary rehabilitation, highlighting the importance of educational campaigns and health literacy initiatives. To address this gap, targeted educational campaigns should be developed and implemented in Lebanon with a multifaceted approach that specifically addresses structural barriers. Collaborating closely with healthcare professionals, such as neurosurgeons, orthopedic surgeons, and nurses, will ensure that accurate, actionable information reaches patients and communities, emphasizing the availability and importance of SCI rehabilitation services. These campaigns should also advocate for policy changes to expand rehabilitation services, particularly in underserved areas, and raise awareness about financial support options, such as insurance coverage and community resources that help mitigate financial constraints limiting access to care [51]. Furthermore, policymakers should prioritize community-based rehabilitation models that bring care and support services closer to individuals with SCI, reducing the need for long-distance travel and making rehabilitation more accessible and affordable [51]. Community engagement efforts through workshops and seminars in diverse settings such as community centers, schools, and workplaces can educate the public about the impacts of SCI and the significance of rehabilitation. These sessions should feature expert presentations alongside personal testimonials to provide both professional insights and lived experiences. Media campaigns across television, radio, social media, and print will reach a broad audience, dispelling misconceptions about SCI and improving health behavior [52], while improving the credibility of information sources by collaborating with trusted healthcare organizations and advocacy groups [53].

For future studies, it is recommended to conduct longitudinal research to assess changes in awareness and knowledge levels over time following the implementation of educational campaigns and interventions. Additionally, qualitative studies could be undertaken to explore in-depth the perceptions, experiences, and barriers faced by individuals with SCI and their caregivers regarding access to and utilization of rehabilitation services in Lebanon. Furthermore, comparative studies across different countries or regions could provide valuable insights into cultural and contextual factors influencing SCI rehabilitation awareness and practices. Moreover, research focusing on the effectiveness of specific interventions, such as community-based rehabilitation programs or technology-based educational initiatives, would be beneficial in informing evidence-based strategies to enhance SCI rehabilitation awareness and access in Lebanon. Lastly, studies evaluating the level of knowledge and practices of health professionals regarding rehabilitation following SCI should be conducted to ensure the delivery of high-quality and comprehensive care to individuals with SCI.

## Conclusion

This study highlights a critical gap in public awareness regarding spinal cord injury (SCI) and its multidisciplinary rehabilitation in Lebanon. The findings reveal a significant lack of understanding of SCI-related complications and the necessity of lifelong medical and rehabilitation care. While some awareness of rehabilitation services exists, the persistent confusion between physical therapy and comprehensive rehabilitation underscores systemic gaps in information dissemination.

Key factors influencing knowledge levels include educational background, occupation, personal connections to individuals with SCI, and prior interactions with rehabilitation specialists. To effectively bridge this knowledge gap, interventions must be tailored to Lebanon’s socio-cultural landscape. Public awareness campaigns should prioritize less-educated populations and rural communities, where misinformation is likely more prevalent. Additionally, healthcare professionals must play a central role in disseminating accurate information through primary care consultations and hospital-based educational programs. Community workshops and media campaigns, particularly through social media and television, can further enhance outreach, while advocacy organizations and educational institutions should collaborate to integrate SCI awareness into school curricula and university programs.

Beyond education, structural improvements are necessary. Strengthening the distinction between physical therapy and comprehensive rehabilitation within Lebanon’s healthcare system can be achieved through policy reforms, standardized rehabilitation guidelines, and interdisciplinary collaboration among healthcare providers. Future research should evaluate the effectiveness of these interventions and investigate the specific barriers individuals face in accessing rehabilitation services. By implementing these targeted strategies, Lebanon can advance public knowledge, enhance support for individuals with SCI, and ultimately improve rehabilitation outcomes and quality of life for this population.

## Supplementary Information

Supplementary Material 1.

Supplementary Material 2.

## Data Availability

The datasets used and/or analyzed during the current study are available from the corresponding author upon reasonable request.
