# Supplementary material for: Public community knowledge regarding multidisciplinary rehabilitation of spinal cord injury in Lebanon: a cross-sectional study
Source: BMC Public Health. 2025 Nov 18;25:4014. doi: 10.1186/s12889-025-25287-3 (PMC12625470; doi:10.1186/s12889-025-25287-3)
Supplement: Supplementary file 1 — Supplementary Material 1. [file 12889_2025_25287_MOESM1_ESM.pdf]

Additional File 1: *Regional Distribution of Participants*

	Population Size*	Population proportion*	Estimated Sample size	Collected Sample
**Beirut**	342000	7	101	104
**Mount Lebanon**	2033000	42	375	376
**North Lebanon**	638000	13	174	175
**Akkar**	324000	7	101	102
**Bekaa**	298000	6	87	89
**Baalbek El-Hermel**	245000	5	73	75
**South Lebanon**	584000	12	163	164
**Nabatieh**	379000	8	114	115
**Lebanon**	4842000	100	1188	1200

**Central Administration of Statistics: about Lebanon [Internet]. 2019 [cited December 23 2023]. Available from: http://www.cas.gov.lb/index.php/about-lebanon-en.*
