# Supplementary material for: Public community knowledge regarding multidisciplinary rehabilitation of spinal cord injury in Lebanon: a cross-sectional study
Source: BMC Public Health. 2025 Nov 18;25:4014. doi: 10.1186/s12889-025-25287-3 (PMC12625470; doi:10.1186/s12889-025-25287-3)
Supplement: Supplementary file 2 — Supplementary Material 2. [file 12889_2025_25287_MOESM2_ESM.pdf]

Dear Lebanese community member,

This study is carried out by a team of specialists in the field of health and rehabilitation, and it is a research aimed at assessing the level of knowledge and awareness of spinal cord injuries and post-injury rehabilitation in Lebanese society. If you agree to participate, we ask you to fill out this questionnaire with complete transparency and honesty to accurately assess the level of knowledge of community members. Your participation in this study is voluntary and takes place after your free informed consent. We guarantee the complete confidentiality of the information, as only the research team responsible for this research will see it while ensuring that your personal information will not be shared. We need to emphasize that we deal with the study data with great responsibility and in a manner consistent with the ethics of scientific research. We thank you for your cooperation and appreciate your time and effort in filling out this form. We hope to answer the questions with complete transparency.

	Question	Options
**Socio-demographic Section**		
1	Age	
2	Gender	Male<br>Female
3	Level of education	- Not educated<br>- Elementary<br>- Secondary<br>- High school<br>- Bachelor’s degree<br>- Higher education
4	Marital Status	Married<br>Unmarried<br>Divorced<br>Widowed
5	Occupational status	Employed<br>Unemployed
6	Occupation	
7	Region of residence	Urban<br>Rural<br>Suburbs

8	Governorate	Beirut<br>Mount Lebanon<br>North Lebanon<br>Akkar<br>Baalbek El-Hermel<br>Bekaa<br>Nabatieh<br>South Lebanon
8	Family Income	100-300$<br>300-500$<br>500-800$<br>>800$
9	Do you live with a person who has a spinal cord injury?	Yes<br>No
10	Do you have any friend or relative who has a spinal cord injury?	Yes<br>No
**General Awareness of Spinal Cord Injury**		
1	**Familiarity of awareness of spinal cord injury**<br>Have you ever heard of spinal cord injury?	Yes<br>No<br>Maybe
2	**Perceived awareness of spinal cord injury**<br>Do you know what SCI is?	Yes<br>No<br>Maybe
3	**Knowledge of the causes of SCI:**<br>What are the causes of SCI?<br>- CVA<br>- Myocardial infarctus<br>- Spinal injury due to road accidents<br>- Infectious disease<br>- Congenital malformation<br>- Spinal injury due to war injuries	Yes<br>No<br>I don’t know

	- Sports injury<br>- Gun Shot injury<br>- Tumor<br>- Diving Accidents	
4	**Knowledge of SCI complications:**<br>SCI complications include:<br>- Quadriplegia<br>- Paraplegia<br>- Hemiplegia<br>- Learning difficulties<br>- Social Problems<br>- Cognitive problems<br>- Self-care difficulties<br>- Sensory problems<br>- Restriction in social participation<br>- Problems in the digestive system<br>- Problems in the urinary system<br>- Sexual Problems<br>- Epilepsy<br>- Chronic pain<br>- Vocational Problems<br>- Respiratory system problems<br>- Visual Problems<br>- Problems with the sense of smell and taste<br>- Life-long disability	Yes<br>No<br>I don’t know
5	**Awareness of the life-long required medical and physical care for individuals with SCI**<br>Does a patient with spinal cord injury require medical and physical management for their whole life?	Yes<br>No<br>I don’t know
**Knowledge and awareness regarding rehabilitation of SCI**		

1	**Self-rated level of knowledge regarding rehabilitation**<br>How do you rate your level of knowledge in the field of rehabilitation?	I don't know<br>Weak<br>Average<br>Good<br>Excellent
2	**Sources of information regarding rehabilitation**<br>What is the source of your knowledge about rehabilitation programs? (Select all appropriate options)	I don't know<br>Healthcare providers<br>During education<br>Social media<br>Personnel research<br>Personal experience with a family member or friend<br>other
3	**Familiarity with health and rehabilitation services for people with spinal cord injuries**<br>Do you consider yourself familiar with health and rehabilitation services or programs for people with spinal cord injuries?	Yes<br>No
4	**Knowledge of the difference between physical therapy and rehabilitation**<br>Rehabilitation treatment for spinal cord injuries is the same as physical therapy	Yes<br>No<br>I don't know
5	**Knowledge of the aims of rehabilitation of SCI:**<br>The aim of rehabilitation for spinal cord injuries includes:<br>- Improve motor function<br>- Financial aid<br>- Environmental modification<br>- Community integration<br>- Independence<br>- Improve the functional role of the individual	Yes<br>No<br>I don't know

	- Transportation<br>- Improve quality of life<br>- Improve speech and expressive functions	
6	**Knowledge of the required different rehabilitation services for individuals with SCI:**<br>Rehabilitation treatment for spinal cord injuries includes:<br>- Physical Therapy<br>- Laboratory Analysis<br>- Pharmacological Treatment<br>- Vocational Training<br>- Chemotherapy sessions<br>- Speech and Language Therapy<br>- Social Work<br>- Psychology sessions<br>- Assistive Devices<br>- Occupational Therapy<br>- Hyperbaric Oxygen Therapy<br>- Mobility aids	Yes<br>No<br>I don't know
7	**Self-reported Knowledge of the multidisciplinary rehabilitation of spinal cord injury**<br>Do you know what comprehensive multidisciplinary treatment in spinal cord injury rehabilitation is?	Yes<br>No
8	**Perceptions towards support for people with spinal cord injury in Lebanese Society**<br>In your opinion, does Lebanese society support people with disabilities in terms of:<br>- Access to rehabilitation care<br>- Accessibility in terms of parking, ramps, working elevators, restroom access, and wide doorways and hallways.	Yes<br>No<br>I don't know

	- Education<br>- Employment<br>- Community integration	
9	**Perceived need for knowledge and awareness about rehabilitation treatments within Lebanese society.**<br>To what degree do you think that members of Lebanese society need knowledge and information about rehabilitation treatments?	Strongly agree<br>Agree<br>Neutral<br>Disagree<br>Strongly disagree
10	**Intent for Information-seeking**<br>How likely are you to seek more information or educate yourself about spinal cord injuries and rehabilitation after this survey?	Very likely<br>Likely<br>Neutral<br>Unlikely<br>Very unlikely

Additional file 2: Questionnaire on the knowledge of SCI and its multidisciplinary rehabilitation
